# Effects of Cognitive Training Programs on Executive Function in Children and Adolescents with Autism Spectrum Disorder: A Systematic Review

**DOI:** 10.3390/brainsci11101280

**Published:** 2021-09-27

**Authors:** Angela Pasqualotto, Noemi Mazzoni, Arianna Bentenuto, Anna Mulè, Francesco Benso, Paola Venuti

**Affiliations:** 1 Laboratory of Observational, Diagnosis and Education (ODFLab), Department of Psychology and Cognitive Science, University of Trento, 38068 Rovereto, Italy; noemi.mazzoni@unitn.it (N.M.); arianna.bentenuto@unitn.it (A.B.); anna.mule@alumni.unitn.it (A.M.); francesco.benso@unitn.it (F.B.); paola.venuti@unitn.it (P.V.); 2 Faculty of Psychology and Educational Sciences, University of Geneva, 1205 Geneva, Switzerland; 3 Laboratory for Autism and Neurodevelopmental Disorders, Center for Neuroscience and Cognitive Systems, Istituto Italiano di Tecnologia, 38068 Rovereto, Italy

**Keywords:** Autism Spectrum Disorder, executive functions, social skills, cognitive training, computerized intervention

## Abstract

Background. Autism Spectrum Disorder is often associated with deficits in executive functions (EFs), which is contributing significantly to individuals with ASD’s difficulties in conducting an independent life, particularly considering social skills. Technologies offer promising opportunities to structure EF intervention programs for children on the autistic spectrum. Methods. This study aimed to review the effectiveness of randomized controlled trials or quasi-experimental studies of EF interventions delivered to children and young people (up to 23 years old) with a diagnosis of ASD. A special focus was dedicated to document the effectiveness of computerized and non-computerized cognitive training on (1) EFs and on (2) ASD symptomatology and social skills. Of 2601 studies retrieved, 19 fulfilled the inclusion criteria. Results. Most of the interventions identified were effective in enhancing EFs and reducing symptoms in children and young people with ASD. Limited evidence is available on their generalization to untrained skills (i.e., social abilities) as well as long-term effects. Conclusions. There is growing evidence for overall effectiveness of EF training, particularly when computerized. However, caution should be taken when interpreting these findings owing to methodological limitations, the minimal number of papers retrieved, and a small samples of included studies.

## 1. Introduction

Autism Spectrum Disorder (ASD) is a neurodevelopmental disorder characterized by socio-communicative impairment and the presence of a repetitive and restrictive pattern of behaviors and interests (DSM-5 [1]) that has a significant negative impact on children’s development [2].

Children with ASD represent a heterogeneous group, given the variability of their symptoms and the presence/absence of comorbidities [3,4,5]. Indeed, the core symptom intensity and severity could vary remarkably among individuals with ASD and could be associated with different atypicalities in the sensory domain, such as hyper- or hypo-sensitivity [1]. As a result, individuals with ASD are likely to struggle in multiple areas, including language, adaptive behavior, and academic performance [6].

Moreover, it is well-known that ASD is frequently associated with various types of cognitive difficulties, which manifest in different ways, such as, for example, executive functioning deficits (for a review, see [7]). Executive functions (EFs)—which, in more general and less reductionist theories, overlap with the concept of Working Memory Capacity and Executive Attention [8]—include different processes that are necessary for individuals to control and update their behaviors [9,10]. EFs have an important role in the adaptation to new environmental stimuli, especially when these require the development of a new behavior in order to be successful. These skills are crucial in mental and physical well-being, academic achievement, and social, psychological and cognitive development [11]. Due to their strong relationship with behavioral regulation, EFs are particularly of relevance for ASD symptomatology and have been increasingly studied in this clinical population [12].

Given the negative impact of executive deficits on the daily functioning of the individuals, especially concerning autonomy, it is important to target executive functioning directly through evidence-based interventions [13]. This is in line with a growing body of research that advocates for an approach of early intervention and that considers neurodiversity and focuses on a variety of cognitive and social domains [14,15].

The goal of the present review was, thus, to systematically analyze the effectiveness of EF interventions in individuals with Autism Spectrum Disorder from an evidence-based perspective. In the next paragraphs, we present a brief overview of the link between EFs and ASD, before reporting our systematic review of the studies on the effectiveness of cognitive training of EFs on children and adolescents with ASD (including RCTs and quasi-experimental studies).

## 2. Executive Functions and Autism Spectrum Disorder

### 2.1. EF Heterogeneity in ASD

Executive functions (EFs) is an umbrella term used to refer to a family of top-down and high-level mental processes that are active when there is the need to concentrate and pay attention, in other words when automatic or instinctual responses would be not desirable or would be insufficient [11,16,17,18].

Traditionally, the study of EF in ASD has focused on discrete EFs domains [7,19,20,21,22,23,24]. These executive processes usually include planning, working memory, attention, inhibitory control, cognitive flexibility, self-monitoring, and self-regulation and are mainly subtended by frontal lobes [25]. However, such approaches are often unsatisfactory since they isolate single executive processes using complex and multifaceted tasks [26,27,28,29]. Indeed, the main criticism to the validity and reliability of EF measures arises from the “task impurity issue”, according to which the neuropsychological tests that are commonly used for EF assessment would measure both multiple EF and non-EF processes [30,31]. In other terms, a multicomponential system is reduced to a simple function [13,32]. For example, the term “working memory” has been incorrectly and reductionistically used to describe one of several executive functions, such as updating (e.g., [9]). Therefore, in order to reduce theoretical-methodological misunderstandings, we prefer more general and less reductionist theories, such as Executive Attention Theory [29,33,34,35], which, according to some authors, is expressed in working memory capacity—WMC [36]. In this theoretical framework, the executive system consists of synergistic interactions between executive, attentive, and memory processes [37].

Conversely, as a result of the reductionist approach predominant in the literature, most of the research on EFs in ASD is characterized by a high heterogeneity of the results [38], encompassing different executive domains [7,20]. Thus, for methodological prudence, the results on EFs and ASD should be reconverted to avoid dangerous statements on the single function, defining instead a more general involvement between different executive processes in interaction [13].

In addition, other factors that may have increased the variability in research on EF performance in ASD include: participants’ age (particularly, in case of mixed age groups), differences in intellectual ability and in their measures (verbal, nonverbal, full scale), as well as different eligibility criteria across studies. In this regard, it has been reported that comorbidity with ADHD affects inhibition abilities in ASD [39], and similarly, comorbidity with stress and anxiety is negatively correlated with inhibition, mental flexibility, and shifting [40].

Moreover, the type of EF assessment that may drive to different results, such as psychometric tests, experimental tasks, and behavioral rating scales, seem to be underpinned by different cognitive mechanisms [41,42,43]. Specifically, behavioral rating scales have been demonstrated to predict better ASD phenotypes and difficulties compared to neuropsychological and experimental measures (e.g., [28,31]) and, thus, seem to have a greater ecological validity [44]. Finally, the format used to administer (computerized vs. traditional), to present the testing materials (visual vs. verbal), and to collect participants responses (verbal vs. motor) may affect EF performance. For instance, ASD participants seem to take advantage of computerized tests [44,45], and visual perceptual tasks [46].

Altogether, these factors may have contributed to the heterogeneity in EF findings in ASD. Nonetheless, recent reviews and meta-analyses have increasingly highlighted the broader influence of executive processes on the ASD behavioral, cognitive, and social phenotype [7]. Interestingly, it has also been hypothesized [47,48] that in ASD individuals, a desynchronization of the Salience Network (SN) system could be responsible for a lack of activation of the Central Executive Network (CEN) in a timely and adaptive manner.

Finally, executive processes have been shown to be associated with social cognition abilities, such as theory of mind [49,50], and with adaptive behavior in daily living skills and socialization domains [51]. It is noteworthy that in adults, difficulties in EFs have been linked to disability [41], mental health [52], and functioning outcomes later in life [53].

Altogether, these findings consistently reported a broad deficit of executive processes in ASD that seems to be stable across development and across ASD phenotypes [11,54,55,56]. This suggests the importance of measuring multiple executive domains using a variety of tasks and tools, as well as of paying attention to their ecological validity, either in research or in clinical settings [13]. Indeed, a careful evaluation of the attentive–executive system in ASD assessment is crucial to support treatments targeted at improving adaptive skills and—more in general—quality of life.

### 2.2. EFs and Social Skills

Research has suggested that EF alterations may explain some core characteristics of ASD [57,58]. For example, it has been suggested that particularly relevant for the social and interpersonal skills are the so-called Hot EFs [59]. Due to their strong relation with behavioral regulation, ‘Hot EFs’ are particularly of relevance for ASD symptomatology and have been increasingly studied in this clinical population [12,52]. According to these authors, Hot EFs operate and guide the behavior when the context or the situation is motivationally and/or emotionally characterized [60]. However, the usefulness of the distinction between hot and cold executive process is still debated as it is not fully supported by neuroscientific and neurophysiological evidence, e.g., [61].

Nonetheless, understanding whether the executive components may contribute to the ASD symptomatology is relevant for developing effective interventions. Indeed, it has been reported that some EF components have an effect on social competence and underlie essential skills for adequate social interactions, such as emotional and cognitive regulation. Fong and Iarocci [62] found that in children with ASD the difficulties in social inferencing and social knowledge were predicted by lower self-monitoring skills, suggesting a different role of EFs in regulating social behavior in ASD subjects. Leung et al. 2016 [63] found that in ASD children the social function was associated with initiation, working memory, planning, organization, and monitoring (i.e., the metacognitive executive processes), suggesting a different relation between metacognitive executive function and social skills in ASD compared to TD children and adolescents. In line with this, metacognitive EFs, such as initiation and working memory [64], as well as initiation and cognitive flexibility [65], were found to play a role also in adaptive social skills (measured by the Vineland Adaptive Behavior Scales).

The relation between EFs and social functioning was supported by the study of Freeman and colleagues, which highlighted that poorer EFs (initiation, working memory, planning, and organization) were associated with reduced engagement with peers and higher playground isolation.

Performance in some specific executive tasks (particularly, the one assessing inhibitory control and cognitive flexibility), emerged to be related to social verbal communication in school-aged children [50]. The relationship between Theory of Mind (ToM) and EFs was reported also in adolescents with ASD [49]. Besides ToM abilities, in adolescents with ASD, higher EFs were found to be associated also with higher empathy (as rated by parent and teacher) [66]. However, further research is needed, as Zimmerman and colleagues [12] found that the impairments in emotion recognition and ToM in adults with high-functioning ASD were not related to deficits in working memory and response initiation and suppression.

Altogether, these studies suggest that specific EFs seem to contribute to different aspects of social competence at different ages, highlighting the importance of targeting specific EF skills to improve the personalization and, hence, the effectiveness of interventions in ASD.

## 3. Cognitive Training of EFs in ASD

Cognitive training exercises have been used in several studies to improve children’s performance thanks to repeated practice on executive tasks [67,68,69,70] (for a review, see [71]). In recent years, a great deal of attention has been paid to improving executive processes, accelerating their development, stopping or slowing declines, and/or addressing deficits [71]. Many methods have been explored and, thus, it has to be noted that the term “cognitive training” is used as an umbrella term for a variety of interventions that often does not provide an accurate or shareable definition. Even within a single treatment, variability of approaches seems common. Nowadays, there are various cognitive training methods available, including downloadable tools, logical games, pencil-and-paper exercises and virtual reality simulations on attention and working memory, to name a few.

Moreover, cognitive training studies with individuals with ASD are very new, and their impact is still an object of discussion [72,73,74,75,76]. Indeed, evidence on cognitive training of EFs is fairly limited and mixed, particularly around the aspects of transfer possibilities, the type of treatment chosen for analyses, and the methodology employed [13]. For this reason, it is important to account for, as much as possible, the numerous variables that might affect behavior and brain plasticity [75,77]. These include participants’ characteristics (e.g., age, comorbid condition) and training characteristics (e.g., duration, intensity, treatment type, setting). Another important feature is the use of technology to support the training activities with ASD children. In fact, Grynszpan et al.’s meta-analysis [78] suggested an overall effectiveness of technology-based training. Children with ASD were found to enjoy the gamification of tasks (i.e., serious games) that provided a safe and secure environment, and in which they commit errors with minor consequences and therefore involve less social anxiety and shame [79]. Furthermore, it has been shown that there might be a galvanizing effect towards technology for ASD children and a steep upper curve in learning though the use of computers [80]. Since then, a growing number of studies have confirmed that the development and evaluation of systems and applications for users with ASD is very promising [79,80]. As technological advancements such as virtual agents, artificial intelligence, virtual reality, and augmented reality become more prevalent, they provide an enjoyable environment for individuals with ASD.

However, studies on the impact of technology-based cognitive training of executive functions have often failed to provide clear and shareable results [76], which has indicated the need for further research. The purpose of this systematic review is, therefore, to document the effectiveness of computerized and non-computerized cognitive training interventions for people with autism by comparing their impact on EFs and on core ASD symptoms, as well as by assessing their methodological quality. Finally, recommendations for future research will be formulated.

### 3.1. Methods

A review of the literature was carried out in accordance with the Preferred Reporting Items for Systematic Reviews and Meta-Analyses—PRISMA [81].

### 3.2. Eligibility Criteria

The research question was formulated using the PICO model [82] and the eligibility criteria were established a priori (see Table 1).

### 3.3. Search

#### Source of Data and Search Terms

To gather the peer-reviewed literature included in the current systematic review, we conducted research using the following online databases from September 2020 to April 2021: PubMed (Title/Abstract); Psyndex, ERIC and Medline via PubPsych (All Text); Web of Science (Title and Topic); Science Direct (Title, Abstract or Author-specified Keywords).

The reference collection procedure was initiated using a search strategy based on the combination of keywords. The full query string is presented below:

(“training” OR “intervention” OR “instruction” OR “program” OR “treatment” OR “remediation” OR “rehabilitation” OR “therapy”) AND (“executive functions” OR “executive function” OR “working memory” OR “inhibition” OR “attention” OR “attentional” OR “attentive” OR “flexibility” OR “planning” OR “shifting”) AND (“children” OR “child” OR “kids” OR “kid” OR “school age” OR “students” OR “student”) AND (“Autism Spectrum Disorder” OR “Autism Spectrum Disorders” OR “ASD” OR “autistic”).

In addition, experts in the field indicated further potentially relevant studies. Finally, a relevant source of information was the reviews and the meta-analysis on the topic, from which the relevant citations to our topic were collected manually.

**Figure 1:**
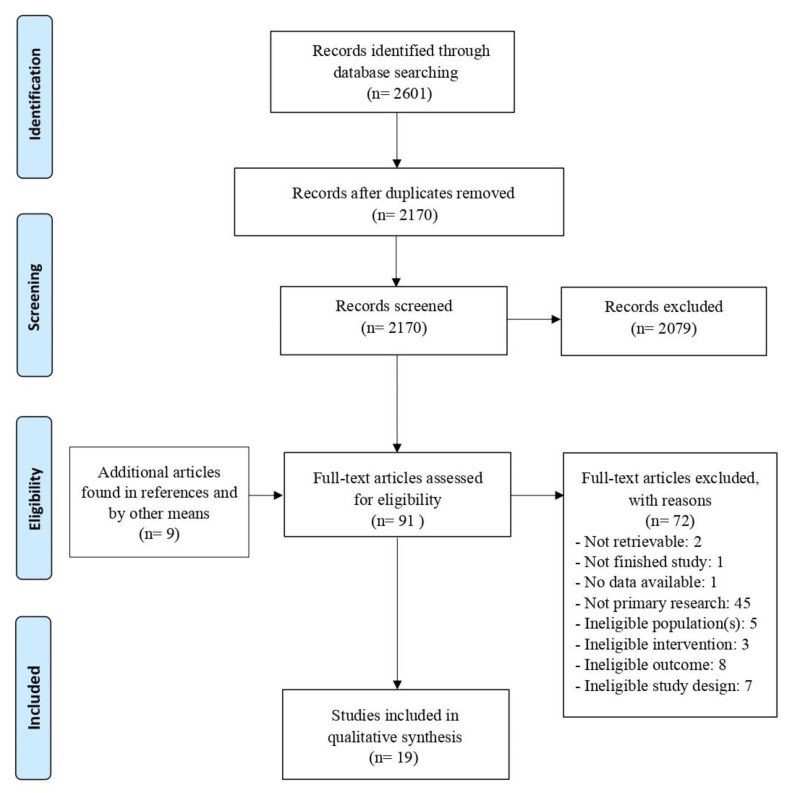
PRISMA flow diagram outlining study selection [81].

This initial search yielded 2601 results from academic journals, dissertations, books, reports, conference materials, and reviews. The PRISMA diagram in Figure 1 describes the search process and provides the rationale behind the exclusion of articles.

### 3.4. Data Selection and Extraction

Titles, abstracts, and full text screening were performed by three independent reviewers.

Disagreements were resolved through discussion with the first author. A consensus was reached among the authors on 72 papers to be excluded, as they did not meet the eligibility criteria, leaving 19 papers for further analysis and coding.

Included studies were then coded for sample characteristics, study design, type of control group, intervention type, intervention target, intervention duration, type of setting and trainer involved, type of pre-/post-measures used, results.

Sample characteristics. The main characteristics of the participants were coded as follows: Sample size: number of participants; Attrition rate: percentage of sample which did not conclude the intervention; Age: mean age (and standard deviation) of the participants, in months. When this was not available, age range was reported; Female rate: percentage of participants that were female, whenever it was reported; Comorbidity: additional diagnosis.

Study design. Randomized controlled trial (RCT); Quasi-experimental (QE).

Intervention type. Intervention approaches used in the article that were coded for categorization in “Computerized” or “Non-Computerized” if the intervention was (or not) primarily delivered on a computer or a tablet.

Outcome. Each result was categorized as Primary or Secondary. Primary: Executive processes. Secondary: a core feature of ASD (i.e., social-communication abilities, restricted/repetitive patterns of behaviors and sensory processing) or a related outcome (i.e., cognitive domains/linguistic/social skills). If outcomes were reported at different time points, follow-up/s were also reported.

### 3.5. Quality Assessment

**Figure 2:**
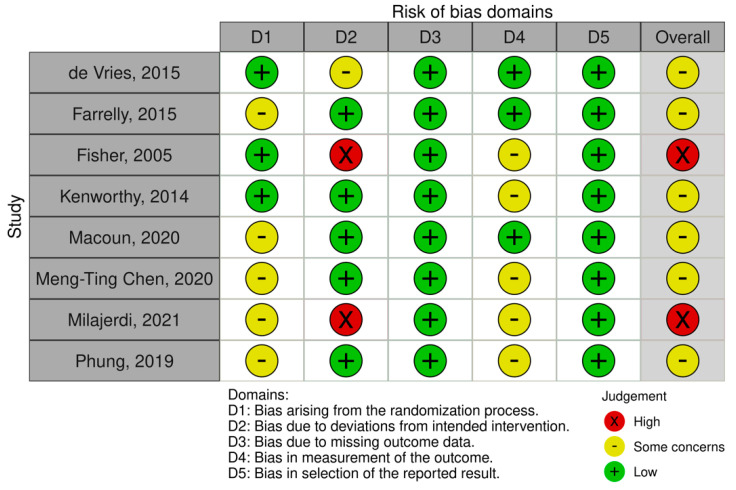
Risk of bias in randomized control trials studies (RCTs) included in this review (green: low bias risk; yellow: some concerns; red: high bias risk).

**Figure 3:**
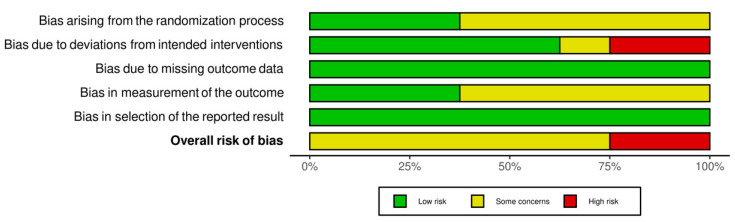
Percentages of risk bias in randomized control trials (RCTs, green: low bias risk; yellow: some concerns; red: high bias risk).

**Figure 4:**
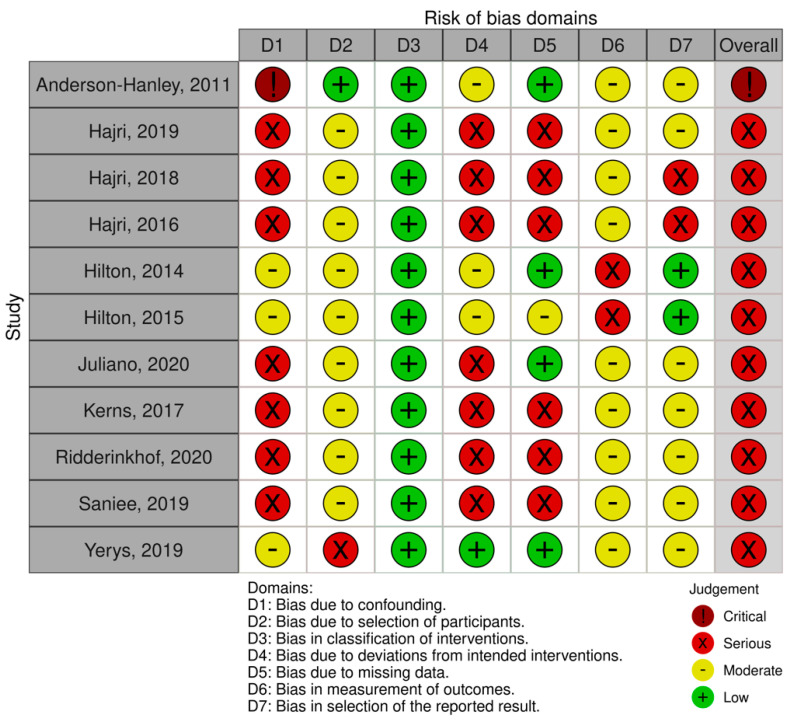
Risk of bias in non-randomized control trials (green: low bias risk; yellow: moderate bias risk; red: serious bias risk; brown: critical bias risk; blue: no information).

**Figure 5:**
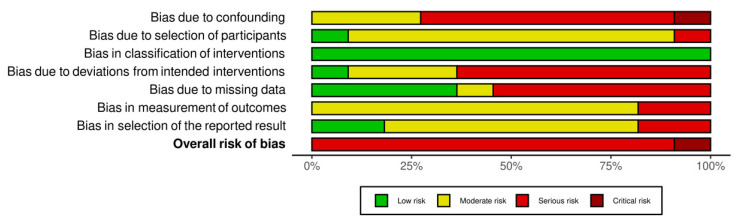
Percentages of risk bias in non-randomized control trials (green: low bias risk; yellow: moderate bias risk; red: serious bias risk; brown: critical bias risk; blue: no information).

Finally, an evaluation of the quality of the results of the reviewed studies was performed (see Figure 2 and Figure 3 for a summary of randomized studies and Figure 4 and Figure 5 for non-randomized studies), according to the Cochrane Collaboration’s criteria [83,84]. Specifically, the following domains were rated: random sequence generation, allocation concealment, blinding of outcome assessment, incomplete outcome data, and selective reporting. For non-randomized studies, a differential code have been used for pre or at-intervention features, covering the following parameters: confounding bias, selection of participants into the study bias, classification of interventions bias. We refer the readers to the Supplementary Information for further details on the parameters used.

## 4. Results

### 4.1. Results of the Search

The systematic gathering procedure performed in the databases returned a total of 2601 papers. Duplicates were excluded, reducing the total number from 2601 to 2170. Initial screening using title and abstract as filter parameters identified 91 studies whose characteristics met the criteria predetermined in the *PICO* question [82]. Nine among them were identified by manual searching the reference lists of 44 articles, including Reviews, Meta-Analysis, Editorials and Dissertations. In accordance with the inclusion criteria, a total of 19 studies were eligible and were included in the qualitative synthesis.

The characteristics of the included studies are presented in Table 2. Due to the heterogeneity of the studies, a meta-analysis of the outcomes was not appropriate. However, narrative results are presented.

### 4.2. Characteristics of Included Studies

#### 4.2.1. Methodological Quality and Risk of Bias

The methodological quality of the included studies was considered problematic in some domains, mainly related to confounding bias, blinding and follow-up data. Even in RCT, many authors did not specify the way in which randomisation was performed, although in most cases the risk of selection bias was never considered critical as allocation concealment was preserved and the characteristics of the participants did not imbalance between the groups. Assessment of the risk of selection bias in non-randomised studies is associated with the presence of confounding factors that may influence treatment decisions. Examples of baseline confounders detected are the level of symptom severity, presence or absence of associated intellectual impairment, socio-economic status, presence of comorbidities, use of medication, and interference of other active interventions during the study. In the majority of the studies, confounding was controlled by restricting the eligibility criteria to subjects who had the same value and type of confounding variables.

Drop out in non-randomised studies was a relevant phenomenon, occurring in 55% of cases. The percentage of subjects lost to follow-up includes subjects who dropped out of the study before the end of the intervention for reasons such as illness, school requirements, scheduling difficulties, or subjects who did not follow the treatment protocol correctly. Furthermore, in some studies, the low number of returned questionnaires led to the exclusion of this information from the analysis. Among the randomised studies, only one reported a high attrition rate, which was treated within the analysis by adopting the intention-to-treat principle [68].

Another qualitative feature that can influence the results and generate questionable interpretations of efficacy is the blinding of participants and outcome assessors, which can be partly remedied by the use of non-subjective instruments and allocation concealment in randomised trials. Of the three cases with the highest risk of bias, two studies did not include enough information about the blinding of the evaluators [85,86], and one study in which the trainer of the intervention coincides with the evaluator of the outcome measures [87]. Overall, in five studies, the authors report the non-blindness of the assessors [67,87,88,89,90] and in ten studies being unclear in their reporting.

#### 4.2.2. Participant Characteristics

The 19 qualitatively assessed studies altogether collected data from 705 subjects with an age ranging from 4 to 20 years. It is worth stating that in four studies, no specific information regarding gender was reported. When reported, the proportion of males within the sample is higher than that of females in all studies with a Male/Female ratio of 5:1, resulting in a percentage of males higher than 80% [68,91,92]. The size of the sample analyzed ranged from 8 subjects [85] up to 100 [68,93].

A total of four studies used a sample size greater than 50 subjects [68,90,93,94]; 50% of the studies reported participants who terminated the intervention before the hypothesized end [67,68,85,86,92,95,96,97,98,99,100] with drop-out percentages ranging from 5,5% [86] to 45,8% [96]. Data lost during the study were in some cases analyzed by applying the principle of Intention To Treat (ITT) that resulted in a percentage of data lost ranging from 6% [94] to 26% [68].

All the participants of the studies included in this review received a certified diagnosis of Autism/Autism spectrum disorder according to the *DSM-IV/DSM-V* criteria that was confirmed using standardized instruments such as *ADOS-1* [88,89,90,93,94], *ADI-R* [67,68,95], *GARS-2* [67,92,95,96], *SCQ* [88,89], SRS [68] and *CARS* [97,98,99]. In 60% of the studies, the average verbal/nonverbal intelligence index was reported, it was measured by *WASI/WASI-II* (Wechsler Abbreviated Scale Intelligence), *WISC-III/WISC-IV* (Wechsler Intelligence Scale for Children), *DAS-II* (Differential Ability Scales), *WRIT* (Wide Range Intelligence Test), *K-BIT* (Kaufman Brief Intelligence Test), *Leiter-R* (Leiter International Performance Scale) and *CPM Raven* (Coloured Progressive Matrices).

#### 4.2.3. Study Characteristics

This review focused on interventions that directly train executive functions throughout several types of behavioral and computerized activities. In this regard, a variety of clinical approaches have been adopted within the various studies, including the cognitive-behavioral model, restorative techniques of the cognitive remediation therapy, mindfulness practice conveyed in martial arts exercise, and cognitive enhancement programs. In addition to this, in light of the most recent innovations in the therapeutic field, we focused on technological advancements that supported training activities with ASD children. Therefore, interventions were categorized into Non-Computerized [89,90,91,93,97,98,99,100,101] and Computerized training programs [67,68,85,86,87,88,92,94,95,96]. A summary of the main characteristics of each study is shown in Table 2.

### 4.3. Characteristics of Non-Computerized Trainings

With “non-computerized” modality we refer to interventions that used cognitive-behavioral techniques such as modelling, reinforcement, scaffolding and self-instruction [90,91,101], cognitive stimulation interventions using Cognitive Remediation Therapy [97,98,99], mindfulness-based programs [93,100], sometimes in combination with martial arts exercises [89].

Most of the interventions were conducted in the school environment, mainly by experimenters or instructors of specific practices such as mindfulness and martial arts; other figures involved were teachers and in one case also parents. Cognitive remediation therapy [97,98,99] was implemented by a therapist in a hospital setting.

The number of sessions falls in a range of 9–28, delivered over a period from 3 to 22 weeks. Exceptions to this include Kenworthy’s study [90], in which 28 training sessions were distributed across a school year, and Fisher’s study [91] in which one session per day was provided for 4–10 days of training in total. The minimum duration of an intervention was 30 min for one session and 1.5 h for the entire intervention—as estimated in Farrelly’s study [101]—while the maximum duration was 19.5 h [89].

### 4.4. Effects on Non-Computerized Training Outcomes

#### 4.4.1. Primary Outcomes

The category of cognitive-behavioral therapies includes several studies that, through a psycho-educational approach, aimed at the gradual and conscious acquisition of functional behaviors [90,91,101]. The application of perspective-taking techniques characterized Fisher’s pilot study [91], in which a group of children involved in set-shifting training (i.e., a EF training) was compared with a ToM training group and a control group that had no intervention. Data were collected after the intervention and at follow-up (6–12 weeks) and included responses to the Card Sort test [91], a simplified version of the WCST that measures cognitive flexibility, and responses of teachers to questionnaires that measure changes related to executive functioning in real life. The results do not reveal any differences between the groups in the questionnaires completed by the teachers. The performance on the Card Sort task showed a similar improvement in the ToM group and in the control group, which was not observed in the EF group.

Promising results came from Kenworthy’s study [90] that evaluated an intervention on EF (Unstock and On Target—UOT) in an ecological context and compared it with a training on social skills. The UOT training includes lessons for resolving concrete experiments, videos, and discussion of different situations to help children use self-regulatory scripts. The lessons of social—communication skills focus on specific skill (e.g physical distance) that are first presented by a didactic class and followed by activities of role-plays and/or games. Both interventions were delivered in the school context by teachers during the school year and reinforced at home through parental support. All the participants were primary school children with ASD without intellectual disability, and were randomly assigned to either the EF intervention group or a social skills’ intervention group. Results showed a significantly greater improvement (medium effect size) in flexibility and problem-solving scores in the UOT group compared to the social skill group, calculated respectively with the WASI Block Design (WASI; Wechsler, 1999) and with the Challenge Task [97]. Furthermore, a greater improvement in the UOT group was also observed in classroom observations and information collected through the BRIEF [102] by teachers and by parents. The classroom-based intervention proposed by Farrelly [101] integrates training aimed at enhancing cognitive flexibility into the school curriculum. Twenty children aged 11–13 years with a diagnosis of ASD were randomly allocated to either a control group or the experimental group. The subjects in the control group did not take part in any intervention, while those in the experimental group participated in three sessions of 30 min each over three weeks. One part of the sessions focused on the social aspects of flexibility using perspective-taking techniques, while another part focused on the cognitive aspects of flexibility using executive tasks such as the WCST [103] and the Stroop Test [104]. One aspect of cognitive flexibility targeted was the social aspect to address deficient perspective-taking and empathy skills. In order to illustrate flexible versus inflexible thinking, the researchers created fictional characters. Then, participants were encouraged to discuss how they might respond to typical social scenarios (shown through cards) in a flexible or inflexible way. Participants were assessed before and after the intervention using the Trail Making Test—TMT [105], an instrument extremely sensitive to cognitive flexibility and with good ecological validity. To minimize the practice effect, the layout of the TMT was modified in the second evaluation. The influence of the intervention in the experimental group results in a larger decrease in time to complete both part A and part B of the TMT than the control group.

Another intervention targeted at EF impairments is cognitive remediation therapy, which was used in Hajri’s studies to replicate in an ASD sample the positive effects that were previously documented in other clinical populations [106,107]. This type of training consists of simple paper-and-pencil cognitive exercises that focus on specific functions such as cognitive flexibility, working memory and planning, with the intention of helping the person to develop the processing strategies. Analysis of differences in pre-post measures showed significant improvements in working memory [97,99] and cognitive flexibility, measured with the Digit Span task [108,109] and the verbal and semantic Fluency task [110,111] respectively. Moreover, post intervention responses to the Hayling Test [112] showed an increase in thinking time that, although not significant, suggests better management of impulsivity. Ridderinkhof [93] used mindfulness with a group of high-functioning ASD subjects to train attentional and inhibitory control. A group of ASD subjects participated in the “MyMind” programme, whose sessions included the teaching of psycho-education and mindfulness techniques, and were compared with a group of TD subjects who had not received any intervention. Before and after the training all participants underwent the Attention Network Test—ANT [113]—prior to the start of the intervention, within two weeks after and two months later. The training lasted 9 weeks and any session had a duration of 1.5 h. The results showed no significant group differences in the Attention Network Test either at baseline or post intervention. Although not significant, the authors reported that ASD subjects showed lower accuracy on executive attention tasks at baseline that improved immediately after the intervention and reached the TD level. At follow-up the observed non-significant improvements were limited only to orienting attention tasks. Alerting attention did not change pre and post treatment. Together with martial arts, mindfulness offered benefits on emotional regulation, working memory and cognitive flexibility [89].

#### 4.4.2. Secondary Outcomes

A few articles considered secondary outcome measures, which reported positive changes following the intervention. Specifically, the use of cognitive remediation therapy helped to maximize participants’ school performance [97,98,99] and decrease their scores on the CARS [97,99]. In this training program, the majority of the activities were conducted individually with the support of paper and pencil materials. In Fisher’s study [91], subjects assigned to the experimental EF condition performed well on ToM tasks at follow-up and showed positive effects in social skills, as indicated by the responses to the items in the teachers’ ad hoc questionnaires created by the researchers to measure EF changes in real life. The results of Kenworthy’s study of 2015 [90] revealed no differences between the two experimental groups, as demonstrated by parent and teacher reports on the SRS [114]. This suggests that EF training led to similar gains compared to specific social skills training, demonstrating the importance of training flexibility to help, for example, the child to better manage his frustration and to interact socially and appropriately with others. These results reflect the benefits of an EF intervention in delivering a trickle-down effect on social skills.

In summary, these results highlighted an increase in EFs, in particular with regard to cognitive flexibility, problem-solving and emotion regulation after non-computerized training. Interestingly, those studies showed that even when the EF training did not have a direct effect on EF improvements, they seemed to have positive indirect effects on social skills in ASD.

### 4.5. Characteristics of Computerized Trainings

Four studies focused on the investigation of intervention programs that combined both motor and cognitive components by engaging participants in the activities of an exergame [85,86,92,94]. In the context of our study, exergames are computer games run on commercial consoles such as the Wii and the Kinect console and that are controlled with body movements. The remainder have investigated the use of serious games and gamified environments to train specific executive abilities [67,68,87,88,95,96].

Most of the interventions were made accessible directly from participants’ homes [68,88,96] or were offered in the school setting [67,85,86,95], where parents or teachers were often actively involved to support performance. In total, five training programs were assisted by an adult [67,87,95,96]; however, each one differed in the level of involvement of the adult within the program. In Saniee’s study [96], for example, computer-based training was integrated with daily life activities using help and promption from mothers. Both Macoun’s [95] and Kerns’s [67] studies involved an research assistant or educator to attend training to learn appropriate metacognitive strategies for supporting and encouraging the young participants during the interventions. Only one study was based in a clinical setting where the training was conducted by a graduate student in clinical psychology under the supervision of a psychologist [87].

Training programs had a varying range of intervention delivery period, number of sessions and total training duration. The range of sessions planned varies between 8 and 24 within 8 to 12 weeks except for Saniee’s study [96], which distributes 4 daily sessions over two months. The duration of training sessions ranged from 2 min [85,86], to 20–30 min [67,88,94,95]; to a maximum of 50–60 min [87,96]. The majority of the interventions lasted between 6 and 12 h in total [67,87,88,95], while three studies focused on extremely brief trainings with a duration of no more than 30 min [85,86,92], and one study [96] has a total of 56 h of training.

### 4.6. Effects on Computerized Training Outcomes

#### 4.6.1. Primary Outcomes

The intent of much training that uses the virtual tool is to take action on multiple executive domains. Sometimes, the integration of activities of daily living and the use of metacognitive strategies allowed for the transfer of skills learned by means of the computer [67,95,96]. Two studies examined the benefits of a serious game on performance in working memory tasks (both in the spatial and verbal domains) and attentional control (i.e., WISC-IV; The Working Memory Test Battery; KiTAP, Test of Attentional Performance for Children). Specifically, Macoun’s study [95] reported significant near-transfer effects in selective attention and visual working memory for the experimental groups compared to a waiting-list group (distractibility attention task; ‘Colored Boxes’ visual-spatial WM task) and in Kerns’ study [67] the ASD sample shows near-transfer improvements in divided attention and verbal working memory (distractibility and divided attention task; the Counting Recall and Listening Recall tasks) compared to sample of subjects with Fetal Alcohol Spectrum Disorders. Another interactive serious game (i.e., Project EVO) was found to be beneficial in improving executive skills in a sample of ASD subjects with ADHD symptomatology [88]. Significant improvements (with medium to large effect sizes) in the TOVA [115], an instrument for screening inhibitory control and attention, in the group who trained with the experimental activity (i.e., Project EVO) compared to the control condition involving an educational word-generation intervention. In Anderson-Hanley’s study [92], participants’ executive skills—assessed through three tasks tapping, i.e., working memory, cognitive flexibility, inhibition—showed significant increases compared to the control only in verbal working memory (i.e., Digit Span Backward [116]). Milajerdi and collaborators [94] examined improvement effects on EF using the WCST [117], whose results confirmed the hypothesis that Kinect training offers greater benefits than traditional physical activity training and a Treatment-As-Usual control group. Intervention programs that focused explicitly on one specific executive function were examined in the other studies, although it is important to emphasize the known possibility of involving other executive processes in accordance with the type of task [13]. Among these works is Chen [87], who proposed a sequence of attentional exercises graded over several sessions in increasingly difficult levels, which required cognitive flexibility to be completed. At the end of the eight-week intervention, participants—compared with a social skills control group—significantly reduced the number of perseverative responses in tests of cognitive flexibility (Trail-Making Test [118]; Wisconsin Card Sorting Test [103]). Saniee et al.’s [96] set-shifting puzzle game not only decreased the production of perseverative responses, but also generalized the skills acquired at the behavioral level, encouraging the subject to shift attention between activities of his/her interest. Interestingly, the improvements were maintained even one month after the end of the intervention. In the study of de Vries and collaborators [68], children who were asked to perform activities prevalent on WM or cognitive flexibility undergo only marginal near-transfer effects compared to the control group (i.e., all the training tasks remained at the low, non-adaptive level) and do not obtain any generalization to untrained skills or maintenance effect in the long term. Benefits offered in terms of generalization emerged in Hilton’s studies [85,86], in which the effectiveness of an exergame was analyzed in a sample of children aged 6 to 18 years. The results showed an improvement in almost all scales of the BRIEF [102], reaching significance levels at the working memory scale and the metacognition index. Cognitive flexibility and inhibitory control skills required during play were transferred to contexts other than that of the intervention (i.e., BRIEF responses).

#### 4.6.2. Secondary Outcomes

More than half of the analyzed interventions assessed the generalization of training improvements to domains other than those directly trained. Specifically, four studies investigated far-transfer effects on social skills [68,87,88,95], finding significant long-term benefits in one case only [87]. Of these, two found no significant differences between the groups [68,88]. Specifically, in Yerys’s study the SSIS was used (The Social Skills Improvement System [119]), while in de Vries’s study the CSBQ (The Children’s Social Behavior Questionnaire; [120]). Six studies reported behavioral changes related to ASD symptomatology [67,92,95,96] and ADHD symptoms [68,88]. Critically, those studies measured the symptomatology using a variety of different tools, and this may have led to different results. For instance, Yerys used the ADHD-RS-IV [121] to measure symptoms of inattention, hyperactivity and impulsivity. In line with the results of the TOVA test, the parent reports confirmed a significant reduction in ADHD symptoms in the experimental group compared to the control group. In the case of Macoun [95] and Kerns [67], the low returns rating data (i.e., BERS-2 [122]; CRS-3 [123]; GARS-2 [124]) did not allow the analysis to be completed. Nevertheless, the interviews with parents, teachers and EAs show an improvement in self-esteem, a greater tolerance of frustration and adequate emotional regulation in response to mistakes. On the other hand, WM-training seems to contribute in de Vries’s study [68] to a slight reduction in ADHD behavior, as can be seen from the scores on the The Disruptive Behavior Disorders Rating Scale (DBDRS, [125]). Participants who trained with the experimental tool in the Saniee and Anderson-Hanley studies [92,96] had better scores on the repetitive and restricted behaviour scale of the GARS [124,126] compared to the control groups.

Finally, two studies reported positive impacts on school performance [67,95]. Similarly to the symptomatology assessment, the measurement of school performance has also been collected using different tools—such as Reading Fluency Curriculum Based Measure [127,128] and Woodcock Johnson III- Math Fluency (WJ-III [129])—leading to a possible heterogeneity in results.

To sum up, the results showed an overall significant improvement in the performance of some executive tasks after a computerized training of EFs. Specifically, the experimental groups improved more than the control groups in the following executive components: attention (both on divided and sustained attention), working memory (verbal and visual) and inhibitory control (decreased number of perseverative responses) through the application of computerized training.

## 5. Discussion

The main aim of this systematic review was to address the emergence in the scientific literature of cognitive training tools that target executive functions in children and adolescents with Autism Spectrum Disorder. An individual with ASD may exhibit a variety of executive deficits and these problems can negatively impact the child’s development, making early identification and targeted intervention crucial [130,131]. Increasing executive skills can positively affect children with ASD’s ability to cope with adult life. Indeed, a timely and effective diagnosis can assist with the planning of targeted rehabilitation interventions in an age group in which significant improvement is most likely to occur.

Our systematic review included randomized controlled trials (RCTs) and quasi-experimental studies and provides up-to-date information on the existent literature on EFs interventions delivered to children and young people with ASD, either through the use of technology (i.e., “Computerized training”) or via more classical paper-and-pencil and behavioral activities (i.e., “Non-Computerized training”). Despite the difficulty in treating ASD comprehensively, several interventions have proved beneficial in improving executive attention, which in turn positively impacts on the quality of life of children with autism and their families. In general, most EF interventions were effective in improving children’s executive skills when compared to control activities or treatment as usual, with some indirect outcomes also over social skills. In a few studies, the evidence indicates that intensive interventions can also be effective in reducing comorbid ADHD symptoms in children and young people with ASD.

The studies on non-computerized training suggests that the greater improvement of the executive functions in ASD are produced by training performed in ecological environments, such as in the school and home—which produced positive effects on shifting, flexibility, problem-solving, and ecological EF measures assessed with BRIEF—and cognitive remediation therapy—which led to improvement in working memory and verbal and semantic fluency [90,97,99,101]. Conversely, interventions based on mindfulness and perspective-taking techniques seem to lead to no or little EF improvement, neither in set-shifting and cognitive flexibility, nor in executive, orienting, and alerting attention [91,93]. Notwithstanding, it is worth noticing that the EF training seemed to have positive indirect effects on social skills in ASD [90,91]. Moreover, the use of cognitive remediation therapy helped to maximize participants’ school performance [97,98,99] and decrease the symptomatology [97,98,99]. Altogether, these results suggest the importance of non-computerized EF training, especially when performed in ecological contests, in supporting the children with ASD to better manage the frustration and to interact with others using socially appropriate strategies.

The extent to which improvements in EF tasks translate to learning is an important issue for educational policies and clinical guidelines [132,133]. So far, little evidence exists on how training effects in specific EFs are transferred into untrained academic skills (e.g., Math grades). Future research should hence investigate this aspect, as it can inform clinical and educational practices.

Regarding the intervention duration, in most of the reviewed studies the training took place over a period of a few weeks (most trials: 2–8 weeks) and ensured a significant improvement in the “quality of life”, suggesting that significant results can be achieved with brief interventions, reducing monetary costs. Notably, most of the studies focused on the analysis of short-term training effects (i.e., 1 week–1 month after the end of the training). Follow-up evaluations after the end of the intervention are pivotal to inform about the maintenance of training gains over time. Therefore, future research should include both immediate and follow-up measures.

Concerning the cost-effectiveness aspect, the use of technology (e.g., virtual agents, artificial intelligence, virtual reality, and augmented reality) seems to support the intervention of ASD children. Computerized training of EFs employed one or more technologies (e.g., tablets, computers, virtual reality) for delivering intervention. The interventions are designed to take advantage of the special interest—often found in the literature [78,134]—that many individuals with ASD have in computer technology. Children with ASD are attracted to digital technologies because they are often predictable and offer clearly defined tasks [135], which minimizes sensory stimulation distractions, provide self-paced usage and require less social interaction [136,137]. Additionally, visual and auditory feedback, such as lights and sounds, provide clear and timely reinforcements [138].

Although digital technologies may be appealing, they are often not thought to produce long-lasting improvements in behavioral performance in children’s real-life activities [139], and some of them can even worsen social behavior. In this regard, results of the studies included in this work suggested that the two types of training may impact individuals’ every day functioning differently: computerized tools produced significant improvements in tasks assessing WMC/executive attention [8,29,140], while non-computerized techniques proved to be more beneficial, even on far transfer measures and, in particular, cognitive flexibility and emotional regulation. Indeed, while technology is a valuable support that should be available to individuals with ASD, EF interventions that are mediated solely by technology may result in limited improvement of social communication or social emotional functioning for children with ASD. In these contexts, the lack of human interaction may result in reduced generalization of the training tools to untrained skills (e.g., social and communicative skills). Although technological supports embed characteristics that make them particularly useful for children with autism, these supports would be more effective if they were integrated into interpersonal interactions, which might include computer-mediated interpersonal interactions, versus replacing interaction partners entirely. This may be especially true when the targeted developmental achievements relate to social abilities. On the other hand, computer-based trials which involved well-trained personnel to support children have been proven to be effective in enhancing not only the trained executive functions, but also in ameliorating self-regulation in daily activities [68,92,95,96]. As a result of strengthening the attentive–executive system, participants were able to sustain longer their focus of attention on the tasks (compared with baseline), often leading to improved planning and organization skills in everyday life [67,92]. Indeed, expert trainers play a major role, especially when dealing with potential emotional or behavioral issues that arise during the intervention. Thus, future research on the effectiveness of computer-based interventions paired with the involvement of expert trainers is not only needed, but also timely. Besides this, greater attention should be paid to the usability and design of technology and computerized training, as this can limit the possible use and access to this type of intervention by individuals with low technological skills [135,138,139].

Although the results of most of the reviewed interventions were associated with a positive improvement in EFs, several limitations characterized the reviewed studies and might impede the results to be straightforwardly applicable in the clinical practice. The major issues are related to the lack of methodological rigor and scarce replication. First, the heterogeneity of the training protocols and outcome measures greatly differ among the studies and prevent us from drawing firm conclusions on training efficacy, as well as the absence of replication studies. Indeed, the results of previous studies might be driven by the number and the durations of the sessions, by the different EF subcomponents that have been trained, or by the used assessment tools which may be too sensitive, or not sensitive enough, to detect EF-related changes. All these limitations impede the clinicians to trust the effectiveness of one or another EFs training and to adopt it within or besides the clinical interventions. In light of that, it would be desirable that future studies sought to replicate the existing findings using more standardized protocols and clinically relevant assessment tools. The improvement in methodological aspects should also regard the increase of the sample size numerosity, analysis of missing data, randomization of subjects, and a comparison of adequate control groups. In addition, statistical analyses should include measures of effect size, corrections for multiple comparisons when multiple measures are used, and the estimate should be published by researchers before beginning recruitment to avoid the phenomenon of “fishing”. Importantly, replication is needed.

Secondly, in addition to standardized EF measures, the outcome measures should consistently include ecological EFs and social skills measures that inform clinicians of the secondary effect of EF training on those capabilities, which are pivotal for self-regulation in daily life and for social interactions.

Third, future research should include ASD individuals with intellectual disabilities, as they represent a great percentage of the ASD population. Indeed, 31% of children with ASD have an intellectual disability (intelligence quotient [IQ] < 70), 25% are in the borderline range (IQ 71–85), and 44% have IQ scores in the average to above average range (i.e., IQ > 85). In addition, the development of interventions for the adult population with ASD also remains necessary. Seltzer and collaborators [141] found that most adults still have difficulty in dealing with the demands of their environment, regardless of their level of impairment. Thus, despite the greater impact of early interventions on ASD symptoms, interventions would also be beneficial for older individuals.

Finally, as mentioned earlier, the functioning profile of children and adolescents with EFs is characterized by a profound heterogeneity (e.g., executive deficits are present in a part of the ASD population). Research should therefore take into account the efficacy of the various interventions on the basis of specific cognitive profiles and severity of symptoms, especially considering the presence of restricted and repetitive behaviors and interests of the participants included in the study. It is likely that the heterogeneity of the results obtained in the studies included in this systematic review could be, in part, explained by the heterogeneity in ASD symptomatology and cognitive profile.

### Clinical Implications

Despite considering the several limitations that emerged from this review, it is important to consider the implications of the results at a clinical level.

Above all, it must be stressed how complex and difficult it can be to carry on training with children and adolescents with ASD. In fact, the use of a computer may improve the sustained attention of the subject, but the repetition of similar exercises may affect the expression of restricted interests. Considering the non-computerized training, the most difficult part is to activate the motivation and interest of the person with ASD; generally, this is possible only after building a positive relationship between the clinician and him/her. Some results regarding the effectiveness of the training, especially in the first part of intervention, may be affected by characteristics of the subjects with ASD, in particular the social difficulties and the activation of the motivation and not for the specific impairment in the EFs. With this in mind, the reviewed studies inform us about the importance of realizing the training in ecological contexts, such as the school and home, and also involving people that are significant for the child, such as parents and teachers. Indeed, besides the therapist, those people well know the strength and the difficulties that characterize the child and, thus, can adapt the training accordingly, maximizing its efficacy. In line with this, a great amount of research has reported the importance of involving parents in the intervention of children with ASD [142,143,144]. Additionally, a wide consensus is present concerning the intensity of intervention necessary to support the development of social-communication and cognitive abilities, and different studies have underlined that parents and school educators have the potential to guarantee more intensive stimulation in the child’s daily life than therapists alone [145,146]. Furthermore, parent-mediated interventions, in which the parent learns appropriate strategies to support the social and communicative development of the child with the therapist during sharing activities [144,147], contribute to better generalization and maintenance of acquired skills. This could be even achieved thanks to participation in the school context. Consistent with this evidence, our review suggests that the positive effect of parents and teachers involved in the treatment may also encompass the EFs.

Moreover, the greater effect of training provided in naturalistic contests, such as in the school and home, highlight the fact that the intervention efficacy is maximized when it is administered not exclusively in therapeutic settings, but also in daily life contexts. In addition, another important result emerging from this review is that the EFs could be improved also at a younger age (i.e., kindergarten children [148,149]). Crucially, the improvement of these functions is positively reflected on the social skills and self-regulation in daily activities, with great potential for ameliorating the adaptation to the life environments and the life quality of both the individuals with ASD and their caregivers. These aspects have a pivotal relevance at the clinical level and should be carefully considered by therapists. Indeed, rehabilitating both the cognitive and the social aspects will offer ASD children the opportunity to train EFs by learning directly from the familiar environment that, in turn, would lead to a higher generalization of positive outcomes. Finally, digital technologies seem to fit well with the ASD characteristics, allowing them to access the computerized training autonomously and to exercise every day without the need of being constantly supported by the presence of a therapist in any session. This is reflected in a decrease in clinical costs and efforts, as well as in an increase in independence and self-monitoring in ASD patients that would promote the generalization of positive training gains. However, briefing and debriefing sessions provided by an expert trainer might be necessary to carry over and generalize skills learned with a computerized tool to real life.

## 6. Conclusions

Most of the interventions reviewed here were associated with a positive improvement in EFs. However, given the publication bias in favor of positive results, intervention studies with negative results are less likely to be published [150]. Therefore, it is possible that the studies included in this review are not a fully comprehensive representation of the EF interventions developed since 2000. In general, although most of the interventions identified have obtained significantly positive results, a suboptimal methodology for many studies and a lack of replication of several training programs impose caution in interpreting the results and make it difficult to draw ultimate conclusions. Although the proof of EF training effectiveness emerging from this review is still inconclusive, the existing body of research supports the importance of conducting timely assessments of executive function and, whenever appropriate, providing targeted interventions [18]. Indeed, the main challenge for the remediation of this disorder is not only to find the most effective remediation programs, but also to precisely select a personalized program for each child with ASD. In fact, the results presented in this review showed a great variability in the effectiveness of training both for the specific EFs and for different groups of children with ASD. For this reason, starting from a careful evaluation of the functioning of the EFs (considering strengths and weaknesses), a clinician will define times, modes and training more suitable for the specific characteristics of the subject with ASD. Nevertheless, we believe that this systematic review could be relevant for researchers and clinicians since it highlights the lack of controlled and randomized clinical trials directly targeting EFs in children and adolescents with ASD and gives important suggestions for improving future research in the field.

## Figures and Tables

**Table 1:** Inclusion and exclusion criteria.

	Eligibility Criteria
Population	Individuals (up to 23 years of age) diagnosed with Autism Spectrum Disorder (ASD).
Intervention	Computerized and non-computerized interventions aimed to target executive functions. The training could be administered individually or in a small group, with different durations and frequencies, in different settings (home, clinics, community, school). In addition, it could be delivered with the support of different figures (psychologists, teachers, parents, speech therapists, health care professionals).
Comparisons	Other types of intervention which did not target executive functions, other interventions that were considered “treatment as usual”, waiting list, no intervention group.
Outcomes	Primary outcomes: Improvement in executive function domains, measured with standardized tests. Secondary outcomes: A core feature of ASD o related cognitive domains/ social skills.
Settings	Any setting (e.g., home, clinics, community, school)
Study design	Randomized control trials (RCTs). If no RCTs were available: quasi-experimental studies or single-group studies were also included. We considered only systematic reviews (SR) or meta-analyses that (1) were included in at least one database (e.g., PubMed); (2) reported the participants inclusion criteria; (3) conducted quality or risk of bias assessment on included studies; and (4) provided a list and synthesis of included studies.
Limits	The SR focused on studies published between 2000 and 2020 with no publication language limit.
Exclusion criteria	Individuals with traumatic brain injuries, primary disorders (sensory, neurological, psychiatric). Editorials, Opinions and Commentaries. Qualitative studies. Single case studies.

**Table 2:** Characteristics of the included studies.

First Author and Year of Publication	Sample Characteristics/ Attrition Rate	Age (Years)	Study Design	Intervention Type	Intervention Target	Intervention Duration	Setting/ Trainer	Outcomes		Pre- and Posttest	Findings
								Primary	Secondary		
Milajerdi, 2021	*n* = 60	6–10	RCT	Computerized “Kinect”	motor skills, EFs	tot hrs = 14 schedule = 3 sess. x week for 8 weeks	N/A/ research assistant	cognitive flexibility	N/A	WCST	Positive
Macoun, 2020	*n* = 23 (2 with ADHD, 2 with tics/sensory)/ 13%	6–12	RCT	Computerized “Caribbean Quest”	WM, inhibitory control, selective attention, and sustained attention	tot hrs = 12 schedule = 3 sess. x week for 8 weeks	school/ research assistant	attention; visual and verbal WM; EF daily life	academic achievement; behavioral symptoms; social skills	KiTAP; SSP and DS; ORF; WJ-III; BRIEF; CRS-3; BERS-2; SSRS; GARS-2	Mixed
Meng-Ting Chen, 2020	*n* = 25 (9 with ADHD)	6–12	RCT	Computerized Comprehensive Attention Training System	sustained attention, sensory selection, response selection and control, attention	tot hrs = 6.6 schedule = once sess. per week for 8 weeks	clinic/ graduate student	cognitive flexibility	social adaptability	WCST; TMT; VABS	Positive
Ridderinkhof, 2020	*n* = 100	8–23	RCT	Non Computerized mindfulness-based program	focused and sustained attention	tot hrs = 15 scheduleNA	N/A/ mindfulness trainer	attention	N/A	ANT	Null
Juliano, 2020	*n* = 29 (18 with ADHD, 11 with Anxiety Disorder, 3 with Sensory Processing Disorder, 1 with Language Disorder)/ 6.8%	10–17	QE	Non Computerized mindfulness-based program	attention, inhibition	tot hrs = 8 schedule = 2 sess. x week for 8 weeks	school/ educator	attention; inhibition	N/A	CWIT; W/DW; CN	Positive
Yerys, 2019	*n* = 19 (with ADHD symptoms)	9–13	QE	Computerized “Project EVO”	attention, cognitive control	tot hrs = 8.3 schedule = 5 sess. x week for 4 weeks	home/ parents	attention; impulsivity; spatial WM; EF daily life	social skills; ADHD symptoms	TOVA; CANTAB; ADHD-RS-IV; BRIEF-2; SSIS	Mixed
Saniee, 2019	*n* = 24	5–7	QE	Computerized “Tatka” + Non Computerized home tasks	set-shifting ability	tot hrs = 56 schedule = 4 sess. x day for 2 months	home/ parents	cognitive flexibility	autism symptoms	MCS; BFRS-R; GARS; ATEC	Positive
Phung, 2019	*n* = 34	8–11	RCT	Non Computerized Mixed Martial Arts	behavioral inhibition, WM, cognitive fexibility	tot hrs = 19.5 schedule = 2 sess. x week for 13 weeks	school/ instructor	behavioral inhibition, WM, cognitive fexibility; EF daily life	N/A	HFT; BRIEF	Positive
Hajri, 2019	*n* = 24/ 33.3%	6–21	QE	Non Computerized CRT	cognitive flexibility, WM, planning	tot hrs = 18 schedule = once sess. per week for 5–6 months	clinic/ therapist	cognitive flexibility; WM	non verbal intelligence; autism symtomps; academic results	SVFT; DSF and DSB; CARS; CMP; academic results	Positive
Hajri, 2018	*n* = 25/ 36%	6–21	QE	Non Computerized CRT	cognitive flexibility, WM, planning	tot hrs = 13.5–18 schedule = once sess. per week for 6 months	clinic/ therapist	cognitive flexibility; WM; planning; inhibition	academic results	SVFT; DSF and DSB; ROCF; HSCT; CAAT; academic results	Mixed
Kerns, 2017	*n* = 23/ 26%	6–13	QE	Computerized “Caribbean Quest”	WM, attention	tot hrs = 12–18 schedule = 2–3 sess. x week for 8–12 weeks	school/ educator	attention; WM; EF daily life	academic results; behavioral symptoms	KiTAP, WMTB-C; SSP and DS; AIMSweb; BRIEF; CRS-3; BERS-2	Positive
Hajri, 2016	*n* = 25/ 36%	6–21	QE	Non Computerized CRT	Cognitive flexibility, WM, planning	tot hrs = 18 schedule = once sess. per week for 6 months	clinic/ therapist	cognitive flexibility; WM;	non verbal intelligence; autism symptoms; academic results	SVFT; DSF and DSB; CARS; CPM; academic results	Positive
de Vries, 2015	*n*= 121/ 26%	8–12	RCT	Computerized “Braingame Brian”	cognitive flexibility, WM	tot hrs = NA schedule = 25 sess. per 6 weeks	home/ parents	cognitive flexibility; WM; inhibition; attention; EF daily life	social skills; ADHD symptoms	Task-Switching; Corsi-BTT; N-back; Stop task; SART; BRIEF; CSBQ; DBDRS-ADHD	Mixed
Farrelly, 2015	*n* = 20/ 10%	11–13	RCT	Non Computerized cognitive flexibility intervention	cognitive flexibility	tot hrs = 1.5 schedule = 3 sess. per 3 weeks	school/ investigator	cognitive flexibility	N/A	TMT	Positive
Hilton, 2015	*n* = 17/ 5.5 %	8–18	QE	Computerized “Makoto Arena”	motor skills, EFs	tot hrs = 0.5 schedule = 3 sess. x week for 5 weeks	school/ graduate student	EF daily life	N/A	BRIEF	Positive
Hilton, 2014	*n* = 8/ 12.5%	6–13	QE	Computerized “Makoto Arena”	motor skills, EFs	tot hrs = 0.5 schedule = 3 sess. x week for 5 weeks	school/ graduate student	EF daily life	N/A	BRIEF	Positive
Kenworthy, 2014	*n* = 67/ 5%	7–11	RCT	Non Computerized “Unstuck and On Target”	physical/mental flexibility, goal setting, planning	tot hrs = 14–18.6 schedule = 28 sess. during 1 school-year	school/ teacher, parent, interventionist	cognitive/behavioral flexibility; planning; EF daily life	social skills	WBD; CT; BRIEF; SRS	Positive
Anderson-Hanley, 2011	*n* = 24/ 8.3%	8–21	QE	Computerized “Cybercycling” or “Dance Dance Revolution”	EFs, exercise behaviors	tot hrs = 0.3 schedule = NA	N/A	WM; switching; inhibition	repetitive and stereotyped behaviors	DSF and DSB; CTT; Stroop task; videotapes according to the RBS of the GARS-2	Mixed
Fisher, 2005	*n*= 27	6–15	RCT	Non Computerized EF training programme	inhibition, set-shifting	tot hrs = 2–4 schedule = one sess. for 5–10 days	school/ investigator	set-shifting; EF daily life	ToM	CST; TMT; ToM and EF questionnaire; FB tasks	Mixed

## Supplementary Materials

Supplementary Information are available online at https://www.mdpi.com/article/10.3390/brainsci11101280/s1.
