# Supplementary material for: Effects of Cognitive Training Programs on Executive Function in Children and Adolescents with Autism Spectrum Disorder: A Systematic Review
Source: Brain Sci. 2021 Sep 27;11(10):1280. doi: 10.3390/brainsci11101280 (PMC8534174; doi:10.3390/brainsci11101280)
Supplement: Supplementary file 1 [file brainsci-11-01280-s001.zip › brainsci-1344419-supplementary.pdf]

Parameters used for the quality assessment of the included studies

For each of the studies, the following dimensions were assessed:

Bias arising from the randomization process (Selection bias):

- random sequence generation, which considers the risk that the allocation of subjects in the experimental and control groups may have occurred in a non-random way, indicating a possible problem in the selection of groups;

- allocation concealment, which evaluates the degree of protection against the risk that the trial operators were aware of the mechanism of random allocation of subjects;

Bias due to deviations from intended intervention (Performance bias):

- blinding of participants and trainers, which take into account the risk due to a lack of blindness of study aims in participants (and their families) and staff (e.g., "placebo effect");

-failures in the implementation of the intervention that could affect the outcome and adherence of participants to the intervention protocol;

Bias in measurement of the outcome (Detection bias):

-blinding of outcome assessment, which evaluates the risk that the study staff responsible for the assessments are aware of group allocation (e.g., enhance risk of detecting improvements after the intervention);

-inappropriate measurement methods or different measurement methods between groups;

Bias due to missing outcome data (Attrition bias):

-incomplete outcome data, which indicates the possible risk deriving from missing data (e.g., under/overestimation of the effects of interventions);

-the proportions of missing data differ between intervention groups;

Bias in selection of the reported result (Reporting bias):

-selective reporting, which assesses the possibility that only a subset of variables is presented (e.g., not presenting measures/subscores for insignificant results).

Note that pre or at-intervention features were coded differently in non-randomized studies addressing the following bias indicators:

Bias due to confounding: selection bias, usually used for randomized trials. Baseline confounding occurs when a prognostic factor determines the assignment of a subject to the experimental group (e.g, age, gender, schooling, impairment level, presence of comorbidities, medication use);

Bias in selection of participants into the study: exclusion of eligible subjects and follow-up times that can affect both the intervention and the outcome;

Bias in classification of interventions: intervention status is well defined for each participant at the start of the intervention, and assignment to the intervention could not be influenced by knowledge of the outcomes.

Glossary of the Abbreviations used for the Assessments tools reported in Table 2

ADHD-RS-IV: the ADHD Rating Scale-IV
AIMS web: AIMSweb curriculum-based measure sure of oral reading fluency
ANT: Attention Network Test (executive, orienting, alerting attention tasks)
ATEC: Autism Treatment Evaluation Checklist
BERS-2: the Behavioral and Emotional Rating Scale
BFRS-R: Behavioural Flexibility Rating Scale-Revised version
BRIEF: the Behavior Rating Inventory of Executive Function
BRIEF-2: the Behavior Rating Inventory of Executive Function 2nd edition
CAAT: Animals Attention Test
CANTAB: The Cambridge Neuropsychological Test Automated Battery
CN: the cancellation subtest (from WISC-IV: Wechsler Intelligence Scale for Children-Fourth Edition)

Corsi-BTT: Corsi Block Tapping Task
CPM: Raven Progressive Matrices
CRS-3: Conners' Rating Scale—Short Version, Third Edition
CSBQ: The Children's Social Behavior Questionnaire
CST: Card Sort Task
CT: the Challenge Task
CTT: the Color Trail Test
CWIT: Color-Word Interference Test (from D-KEFS: the Delis-Kaplan's Executive Function System)
DBDRS-ADHD: The Disruptive Behavior Disorders Rating Scale
DSFand DSB: Forward and Backward digit span tasks
FB tasks: false belief tasks test (e.g., penny hiding deception task, reading the mind in the eyes' task)
GARS: Gilliam Autism Rating Scale
GARS-2: Gilliam Autism Rating Scale-Second Edition
HFT: Hearts and Flower Test
HSCT: the Hayling Sentence Completion Task
KiTAP: Test of Attentional Performance Children's Version
N-back: N-back task
ORF: Oral Reading Fluency Curriculum Based Measure
ROCF: the Rey-Osterrieth Complex Figure
SART: the Sustained Attention Response Task
SRS: Social Responsiveness Scale
SSIS: the Social Skills Improvement System
SSP and DS: Spatial and Digit span tasks (from WISC: Wechsler Intelligence Scale for Children)
SSRS: the social skills rating system
SVFT: verbal and semantic fluency tests
Task-Switching: Gender/Emotion -and Number-gnome switch-task
TMT: the Trial Making Test
TOVA: Test of variables of attention
VABS: Vineland Adaptive Behaviour Scales
W/DW: the W/DW subtest (from the TEA-Ch: The Test of everyday attention for children)
WBD: the WASI Block Design (from WASI Wechsler Abbreviated Scale of Intelligence)
WCST: Wisconsin Card Sorting Test
WJ-III: Woodcock Johnson III- Math Fluency
WMTB-C: the Working Memory Test Battery

Training characteristics of the included studies

Milajerdi, 2021
Kinect is a virtual exercise program that allows people to follow their body movements as they engage in interactive tasks requiring motor and cognitive skills. The study aimed to compare the effects on EFs of a Kinect group with a group involved in traditional physical activity training and a TAU control group. The intervention was delivered three times per week (35 min. per session) for eight consecutive weeks by a research assistant responsible for teaching the child the correct movements and giving feedback on performance.

Macoun, 2020
The computerized training is a serious game using a process-specific approach that consists of five hierarchically structured, self-adjusting mini-games focused on WM, inhibitory control, selective

attention, and sustained attention.A research assistant delivered the CQ ("Caribbean Quest") intervention in a one-to-one format during school hours. Participants had 24 sessions of 30 minutes each scheduled three times per week for eight weeks total of intervention (tot hours: 12). The role of the adult trainer was to support the child during gameplay sessions by teaching appropriate metacognitive strategies to facilitate participation, as well as to increase the generalization of the effects of the intervention.

Meng-Ting Chen, 2020
CATS training has been designed to enhance executive functions with digital tasks involving four attentional components. Tasks required the children not just the attentional skills as well the ability to inhibit irrelevant stimuli and the flexibility to change rules as activities become difficult. The intervention compared with an experimental control condition involving social activities was given once a week for eight consecutive weeks (each session lasted 50 min). Participants in both experimental conditions were paired and received and followed instructions from a trainer.

Ridderinkhof, 2020
Children parallel their parents participated in the Mymind program that provided psychoeducation sessions and mindfulness and relaxation exercises. The intervention led by a mindfulness trainer included lessons in small groups in which participants learned to apply relaxation and self-regulation techniques to stressful situations. The half-hour-long sessions were delivered once a week for nine weeks.

Juliano, 2020
The study proposes a mindfulness program during school intending to increase the skills of prepotent response inhibition, interference control, and selective attention in a group of high functioning ASD children. The intervention consists of 16 sessions focused on different aspects of mindfulness, such as mindful breathing, bodies, listening, thoughts, and emotions, as well as learning and practicing a corresponding skill. Participants dividing into groups guided by a mindfulness instructor undertook two sessions (30 min each) per week for eight weeks.

Yerys, 2019
Semi-randomly the recruited subjects were allocated in the experimental group receiving the multi-tasking digital treatment or in an active control group involved in a non-multi-tasking educational treatment. The multi-tasking digital treatment is a video-game intervention targeted to cognitive control that requires children to rapidly switch between a perceptual discrimination attention/memory task and a continuous visuomotor driving-type task. Participants are given an iPad for four weeks with the app installed and asked to complete 20 sessions with a frequency of 5 sessions per week, each lasting 25 minutes per day. The degree of difficulty was automatically adapted to the child's level and gradually re-calibrated based on their progress, whereas task engagement is monitored remotely by an investigative team.

Saniee, 2019
The intervention included a computer game and home tasks to extend improvement of flexibility also at the behavioral level. The digital part consists of a puzzle game whose solution requires adapting reasoning to the changing rules. The training continued at home with the support of the mothers accustoms the child to change between interesting or uninteresting activities trying to overcome his resistance. Subjects receive both trainings at home for two months dedicating 75 minutes to activities of daily living and 60 minutes to the computerized task.

Phung, 2019
The MMA (mixed martial arts) program targets three core EFs: behavioral inhibition, working memory, and cognitive flexibility. The 13 intervention sessions conducted by martial arts instructors included increasingly difficult fighting techniques, that required the engagement of executive functions such as behavioral inhibition to dose force, working memory to remember the rules associated with different body movements, cognitive flexibility to manipulate mentally acquired information to accommodate new ones. Each class session included no more than 12 ASD children assisted for 4 hours per month by TD peers who were martial arts students. Participants in total completed 26 45 min intervention sessions delivered twice a week for 13 weeks.

Hajri, 2019 (-2018, 2016)
Cognitive Remediation Therapy is a paper/pencil method administered face-to-face with the therapist/child, including a set of exercises categorized into three modules (cognitive flexibility, memory, planning). Each module was addressed in six to eight sessions of 45 minutes each over approximately six months, totalizing an average of 22 intervention sessions per participant.

Kerns, 2017
"Caribbean Quest" is a serious game using a process-specific approach that consists of five hierarchically structured, self-adjusting mini-games focused on WM, inhibitory control, selective attention, and sustained attention. An assistant educator under the supervision of a research assistant delivered the CQ ("Caribbean Quest") intervention in a one-on-one format during school hours. Participants completed 12 hours of training three times a week over 10 to 12 weeks. The role of the adult trainer was to encourage the child to cope with game difficulties and learn how to monitor performance through the use of metacognitive strategies. During the intervention, the support of the adult gradually decreases, leaving the child to be increasingly independent in the management of performance.

de Vries, 2015
Braingame is a video game consisting of visual-spatial memory, inhibition, and cognitive flexibility tasks. Each participant performed all tasks but with different adaptive levels depending on the experimental condition in which they were allocated (e.g., WM training, flexibility training, or mock-training). During the six training weeks (25 sessions), the parents could support the child in playing.

Farrelly, 2015
The classroom-based intervention proposed by Farrelly integrates training aimed at enhancing cognitive flexibility into the school curriculum. One part of the sessions focuses on the social aspect of cognitive flexibility with activities that aim to encourage the participant to think flexibly in the face of social situations that might occur at school or home, first by identifying with two fictional characters and then applying the ideas to themselves. The other aspect of cognitive flexibility considered is cognitive flexibility, which was addressed using two rule-switching tasks (WCST and Stroop Test). The intervention group received three, 30-minute intervention sessions over the course of three weeks, whereas the control group did not undertake any intervention.

Hilton, 2015 (-2014)
Makoto exergame is a triangular arena with three towers spaced 6 feet apart on its three corners. Each tower has 10 lights, which illuminate with an associated tone in random order. The goal was to identify and tap the light as rapidly as possible before time ends, training speed, attention, and

physical fitness. Participants could choose the frequency of the intervention. They typically completed three 2-minute sessions per day, totalizing 6 sessions per week over approximately 8 weeks.

Kenworthy, 2014
The intervention delivered at home and at school aimed to train planning, flexibility, and insistence on identity using self-regulation scripts, visual aids, and verbal aids. The comparison intervention included didactic lessons and hands-on role-plays activities focused on social-communication skills.Children received approximately 28 30-40 min small group intervention lessons conducted by school teachers then reinforced at home with parental support.

Anderson-Hanley, 2011
The study uses two exergames as enhancement training to investigate the effectiveness of the combination of cognitive and physical exercises on the executive system. Subjects participated in the initial session in a placebo condition, asking them to watch a video on TV and after one week in the exergame session, both lasting 20 minutes**.**

Fisher, 2005
The study compared a group of children subjected to a set-shifting intervention with a ToM training group and a control group subjected to no intervention. Intervention encourages the child to think through analogies and change perspective according to the scenario represented on the paper that could include some element on ToM that indirectly trained. Training programs were administered individually, lasting for 25 minutes per day for 5-10 days.
